# Pulmonary Emphysema in a Cow

**Published:** 1901-01

**Authors:** C. H. Sweetapple

**Affiliations:** Ontario Veterinary College, Toronto, Canada


### REPORTS OF CASES.

### PULMONARY EMPHYSEMA IN A COW.

By C. H. Sweetapple, V.S.,
ONTARIO VETERINARY COLLEGE, TORONTO, CANADA.

Among the numerous post-mortem examinations that I am accustomed to make on cattle for butchering purposes in the city of Toronto, Canada, for the health department of the city, the following case may be of interest to your readers, as so marked a case of pulmonary emphysema in the ox tribe—the animal otherwise in perfect health—is probably seldom met with.

A short time ago I was called to the Toronto cattle market to examine a fat cow. Her respirations were very frequent, but *not abdominal.* Temperature, 102° F. Pulse, normal. Healthy moisture on the nose. Auscultation revealed no mucous râle in the trachea or bronchi; in fact, no abnormal sounds whatever that I could discover. On percussion of the thorax I fancied that her right side was a little more resonant than the left ; but there are certainly better places for auscultation and percussion than a crowded cattle market. There were no febrile symptoms whatever. The cow was ordered to be retained for a few days and then to be butchered under inspection, as it was probable that by rest and quietude for a short time her respirations might improve.

Four days afterward I again examined her at the slaughterhouse. I found no change in the respirations. They were quite as frequent as before, and all the other physical signs of health as previously seen, and I was told that the appetite and rumination, as well as the evacuations from the bowels and kidneys, were normal.

The animal was then butchered in the ordinary way, the viscera removed and also carefully examined. There were no adhesions, abnormalities, or pathological changes of any kind that I could discover, with the exception that the greater part of the large lobe of the right lung was greatly distended with air (interlobular emphysema), the distention being much greater than in the same condition in the horse, owing to the well-known fact of the much greater amount of areolar tissue in the lungs of the ruminant. The carcass was well nourished and fat.

The only history I could learn of the case was that the farmer had said that the animal's breathing had been as described "ever since several months ago, when she had been chased out of a field of standing corn by his dog. Her stomach was fully distended with green food at the time, and she had been pretty well chased."

